# Fluidized Bed Drying of Pumpkin (*Cucurbita* sp.) Seeds

**DOI:** 10.3390/foods8050147

**Published:** 2019-04-30

**Authors:** Saheeda Mujaffar, Sheena Ramsumair

**Affiliations:** Food Science and Technology Unit, Department of Chemical Engineering, The University of the West Indies, Saint Augustine, Trinidad and Tobago; sheena_r14@hotmail.com

**Keywords:** fluidized bed drying, pumpkin seeds, pumpkin seed powder, thin layer modelling, drying kinetics

## Abstract

Pumpkin seeds are a major agricultural waste from the fresh-cut produce industry. The objective of this study was to investigate the drying behavior of untreated, whole pumpkin seeds in a fluidized bed dryer at 50–80 °C (2.87 m/s), with a view to producing a high-quality pumpkin powder from dried seeds. Seeds were dried at 50–80 °C to an average equilibrium moisture value of 0.035 to 0.006 g H_2_O/g DM (3.4 to 0.6% wb). Drying occurred in the falling rate period only and drying rate constants ranged from 0.0226 to 0.0900 1/min with corresponding diffusivity values for the first falling-rate period ranging from 4.68 to 18.63 × 10^−10^ m^2^/s. The activation energy (*E_a_*)—for the first falling rate period was determined to be 43.9 kJ/mol. Of the nineteen thin layer models tested, the Alibas model could be successfully used as a general model to predict the Moisture Ratio (*MR*) data for all temperatures investigated. After drying, seeds were blended to produce powders, which were found to be high in fat, crude protein and fiber.

## 1. Introduction

Pumpkin belongs to the genus *Cucurbita*, family Cucurbitaceae, with *Cucurbita maxima* being the species most commonly found in the Caribbean region. The most used part of the pumpkin fruit is the flesh, which is used as a vegetable in food preparations. Pumpkin seeds are rich in oil, protein and fiber and are a valuable source of minerals [1], yet despite their high nutritional value, the seeds are usually discarded and are therefore considered a major agricultural waste of the fresh cut produce industry. The most common use of pumpkin seeds, primarily from the hull-less seed type pumpkin varieties, is as a ready-to-eat snack. The seeds are sold in the raw, dried or roasted form and can be salted or flavored with various spices. For the varieties of pumpkins that produce seeds with hulls, the seeds are first subjected to a hull-removal process. 

There is a growing interest in the production of pumpkin seed powders, which are made by first drying the seeds and then grinding the dried seeds to produce a fine powder, sometimes called a “flour” due to its appearance. For hull-less seed varieties, the whole seeds may be used in the preparation of the flour or for hulled-seed varieties, the hull can be manually removed prior to drying or roasting [2,3,4,5,6]. Due to the difficulty in removing the hulls, seeds are boiled in water for 1 h prior to removal, and in some cases, the dried seeds are subjected to a de-fatting process using n-hexane [2,6,7,8,9]. 

Previous works have focused on the nutritional evaluation and functional properties of the seed powders and the use of the powder in various food applications, especially in bakery products such as cookies and biscuits and in other products such as weaning mixes [2,3,4,5,6,7,10,11,12]. Like the seeds, pumpkin seed powders are reported to be a good source of protein, and depending on the seed type and processing method, an excellent source of fiber and oil. 

While drying of seeds for the preparation of powder has been carried out using sun drying, the most common drying method is oven (tray) drying (60 °C, 4–24 h) [2,4,8,11,13,14]. The pioneering research works on the drying characteristics of pumpkin seeds have focused primarily on the drying of salted (25% (*w/w*) NaCl, 1 h) seeds with a view to optimizing the drying process to produce a snack [13,15,16]. Drying curves for salted seeds of the hull-less variety (*Cucurbita styriaca*) dried at 40–60 °C (0.8 m/s) in a laboratory scale hot air dryer, as well as in the open sun and in a solar tunnel dryer have been presented [13]. Effective diffusivity values (*D_eff_*) and activation energy (*E_a_*), were determined and the Moisture Ratio (*MR*) data modelled using four well-known drying models. The drying kinetics of salted, whole pumpkin seeds (*Cucurbita* spp.) dried in a tray dryer and a fluidized bed dryer at 60–80 °C (0.23–0.28 m/s and 1.8 m/s) was investigated [15]. The Moisture Ratio (*MR*) data was modelled using six thin layer models and the effective diffusivity values (*D_eff_*) were found to be similar to those presented by the previous author for de-hulled seeds, possibly due to the higher temperatures and air velocities used. A comparison of tray and fluidized-bed drying of whole, salted seeds was continued at temperatures of 70–90 °C and air velocities of 2.0–4.0 m/s [16]. Drying conditions of 70 °C, 2 m/s was recommended for salted seeds.

Fluidized bed drying is a rapid drying method suitable for particulate (20 µm to 5mm) and heat-sensitive food pieces, including seeds [17]. By forcing hot air through a bed of particles, the particle bed assumes a fluid-like state (hence the term fluidization). High heat and moisture transfer are achieved due to thorough mixing of the material resulting in uniform temperature distribution and increased surface area for drying. While there is potential for use of this type of drying for pumpkin seeds, too rapid drying can, however, result in seed discoloration and undesirable changes in seed texture, as reported for salted pumpkin seeds [16]. 

The production of a pumpkin seed powder is an attractive option for the Caribbean Region, where pumpkin seeds are discarded. The varieties of pumpkins that are common to this Region contain seeds with hulls, and hull-removal is a tedious process. No works have been reported on the drying characteristics of whole (untreated) pumpkin seeds using fluidized bed drying method, for the purpose of producing a high protein, high fiber, pumpkin powder. The objective of this study is therefore to investigate the drying behavior of pumpkin seeds dried in a fluidized bed dryer and report on selected quality characteristics of the pumpkin seed powder.

## 2. Materials and Methods

### 2.1. Preparation of Seeds

Pumpkin seeds (*Cucurbita* sp.) were obtained from a local packinghouse facility as a waste product from the processing of a minimally processed pumpkin product. The seeds were cleaned and sanitized using 200 ppm chlorine solution and left to dry at ambient room temperature (30 ± 2 °C) for five hours to remove any residual moisture from the washing process. This step was carried out to prevent sprouting of the seeds in refrigerated storage due to the presence of excess moisture. Seeds were then placed into plastic bags and stored in a refrigerator at 4 °C [13]. 

### 2.2. Drying Equipment

**Figure 1:**
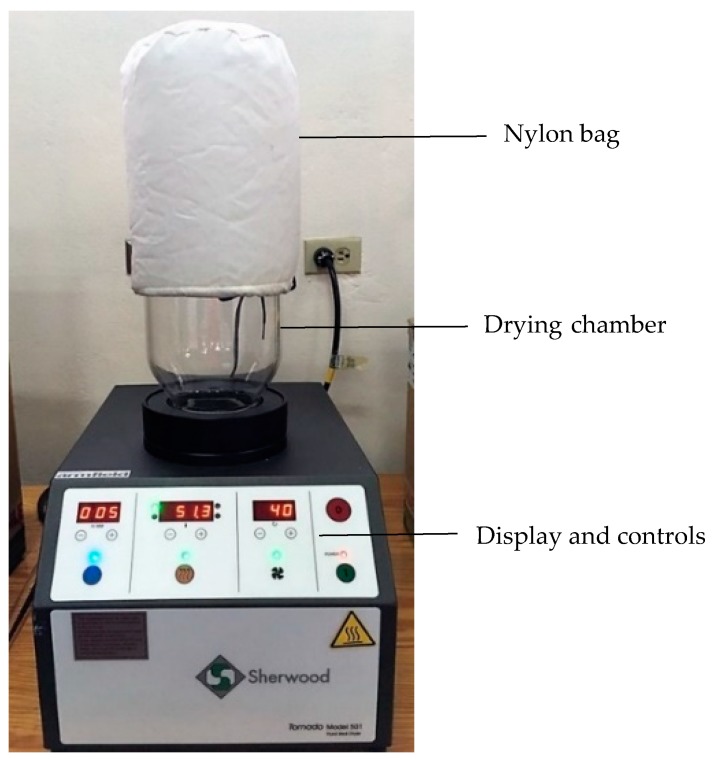
Sherwood Scientific Tornado M501 Fluid Bed Dryer.

Fluidized bed drying of the pumpkin seeds was performed using a Sherwood Fluid Bed Dryer (Sherwood Scientific Ltd., Cambridge, UK) shown in Figure 1. The dryer consisted of a base unit with a timer, temperature (20–199.9 °C) and velocity (0–127) display and control, a 2 L glass tub with inlet filter and a nylon bag. Velocity/blower settings corresponded to a range in air velocity from 0.35 to 6.70 m/s and ambient air drawn through the inlet air filter by a centrifugal blower was heated by a 2 kW electrical heater.

### 2.3. Drying Procedure

Seeds were removed from the chiller about one hour before drying to reach ambient temperature before drying. The fluidized bed dryer allowed to set to the desire temperature and air velocity for 15 min prior to loading of the samples into the pre-weighed glass jar. For each drying run, sample size averaged 50 g (approximately 150 seeds), which corresponded to a static bed depth of 3 cm [15]. During drying, the jar with the seeds was removed at 5min intervals, quickly weighed (0.01 ± 0.005 g) using an Explorer Pro Balance, Model EP2102C (Ohaus Corporation, Parsippany, NJ, USA), and returned to the base and seeds were dried until constant weight was achieved. All drying runs were carried out in duplicate. After each run was completed, the dried pumpkin seeds were left to cool, then ground to a course powder using a heavy-duty blender -51BL30 (Waring Commercial, Torrington, CT, USA) to produce a powder. Ground samples were stored in polyethylene bags in a desiccator until ready for analysis. 

### 2.4. Drying Variables

Drying temperatures of 50 °C, 60 °C, 70 °C and 80 °C were used as described for fluidized bed drying of squash seeds [18]. Based on preliminary work by the authors [19], the minimum velocity in which full fluidization of the seeds occurred was 2.39 m/s (blower setting 40) that is, *u_min_* for 50 g samples was ≤2.39 m/s. Analysis of moisture changes and drying rates revealed that the effect of increasing the air velocity from 2.39 to 2.87 m/s did result in a small improvement in drying rates, which were most noticeable at 50 °C. Increasing air velocity above a setting of 50 (2.87 m/s) caused rapid blowing of the seeds which got caught in the cover sack, especially as they dried and became lighter. Therefore, a blower setting of 50 (2.87 m/s) was subsequently used for all experiments. Fluidization of 40 g samples of whole squash seeds was reported to occur at an air velocity of 2.51 m/s [18]. 

### 2.5. Analytical Methods

All methods were carried out in triplicate. The moisture content of the fresh and dried seeds was done using a Halogen Moisture Analyzer HB43-S (Mettler Toledo-AG, Zurich, Switzerland) set at 130 °C, which was determined to be the maximum temperature that could be used without burning of the leaves. Water activity (*a_w_*) of the fresh and dried seeds was measured using an Aqua Lab CX-2 1021 water activity meter (Decagon Devices Inco., Pullman, WA, USA). Surface color of the powders was measured using a CR-410 Choma Meter (Konica Minolta Sensing Americas, Inc., Ramsey, NJ, USA). Hunter values (L*, a*, b*) were recorded and Hue angle (°) and Chroma calculated [20]. Fat (%) was measured using the AOAC 920.39 Randall Method for Fat Analysis [21], using petroleum ether. Protein content (%) of the pumpkin seed powders was determined using the Kjeldahl method [22] and crude fiber content (%) of the defatted pumpkin seed powders was determined [21] using a fiber extraction unit (Labconco Corporation, Missouri, KS, USA). Defatting of the seed powder was done using a Model 1021 Foss Analytical Ab Cold Extraction Unit (Foss Analytical Ab, Hoganas, Sweden). 

### 2.6. Powder Physical Properties

Selected physical properties of the pumpkin seed powders were also determined. Sieve analysis was done using a DuraTap Testing Sieve Shaker (White Stone, VA, USA) using sieve numbers 6 through 100 (aperture sizes 3.36–0.149 mm) and the percentage of sample retained on each sieve calculated. Water solubility (%) and water adsorption capacity (%) were determined using methods described for pumpkin powder [23]. Bulk and tapped density of the pumpkin seed powders was carried out and the Hausner ratio (*HR*) and Carr index (*CI*) calculated [24]. 

### 2.7. Data Analysis

The analysis of the drying data was done as previously described [25,26]. Drying curves (moisture content g H_2_O/g DM versus time t) and drying rate curves (rate g H_2_O/g DM.min versus average moisture content g H_2_O/g DM) were constructed. Moisture Ratio (*MR*) curves (*M-M_e_/M_o_-M_e_*) were constructed based on the analytical solution of Fick’s Law for an infinite slab assuming uniform initial moisture distribution and negligible external resistances. Drying rate constants (*k*) were determined from the initial straight-line portions (60 min) of plots of ln *MR* as a function of drying time (*t*) and effective diffusivity values calculated. The temperature dependence of the *D_eff_* values and the activation energies were estimated from plots of ln *D_eff_* versus *1/T* using an Arrhenius type equation. Ertekin and Firat [27] noted that thin layer modelling could be applied to food particles that are freely suspended in the drying air. Nineteen (19) empirical and semi-empirical thin layer models (Table 1) were applied to the *MR* data [28] and the performance (fit) of the models was assessed using the coefficient of determination (*R*^2^), root mean square error (*RMSE*), and the chi-square statistic (*χ*^2^) [13,15,18]. One-way ANOVA was carried out to ascertain the effect of temperature on the equilibrium moisture content, water activity, time to equilibrium, and color values as well as the physical attributes and results of the proximate analyses [29].

## 3. Results and Discussion

### 3.1. Moisture and Water Activity (a_w_)

Initial moisture and water activity content values of the pumpkin seeds averaged 0.486 ± 0.010 g H_2_O/g DM (32.71 % wb) and 0.917 ± 0.020, respectively. As expected, seeds experienced a loss in weight and decline in moisture content as drying proceeded, and the higher the temperature the greater the rate of decline. The maximum weight loss (over original weight) averaged 32% for seeds dried at 60, 70, and 80 °C, respectively, while the maximum weight loss for seeds dried at 50 °C averaged 27%, indicating that seeds dried at 50 °C did not lose moisture to the same extent as those dried at higher temperatures. 

Table 2 shows that the total drying time to attain equilibrium moisture content, equilibrium moisture values and final water activity values of dried seeds were significantly (*p* ≤ 0.05) affected by drying temperature. Increasing the temperature from 50 °C to 60 °C resulted in an 18% decrease in drying time while further increasing the temperature to 80 °C resulted in a 40% decrease in drying time, compared with seeds dried at 50 °C. Seeds were dried at 50–80 °C to an average moisture value of 0.035 to 0.006 g H_2_O/g DM (3.4 to 0.6% wb). The equilibrium moisture values obtained in this study were lower than the 0.053 g H_2_O/g DM (5.0% wb) moisture value reported for hull-less pumpkin seeds dried in a tray dryer at 40–60 °C [13], indicating that drying was more efficient using the fluidized bed drying method.

**Figure 2:**
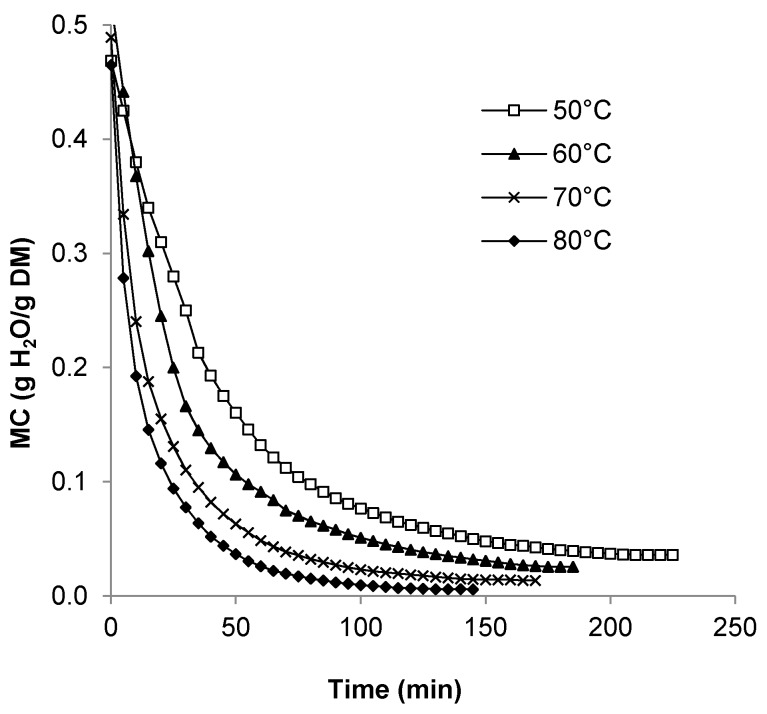
Drying curves for pumpkin seeds dried at different temperatures at a fixed air velocity of 2.87 m/s.

Drying curves for seeds dried at 50 to 80 °C at an air velocity of 2.87 m/s are illustrated in Figure 2. The higher the temperature, the greater the decline in moisture values, especially noticeable during the first 50 min of drying. The increase in decline in moisture content versus time with increasing temperature is a common observation of many food dehydration studies and was also shown for salted, hull-less pumpkin seeds dried in a tray dryer at 40–60 °C [13]. To achieve a minimum of approximately 4–6% (wb) moisture which appears to be the most commonly reported moisture value for dried seeds, seeds can be dried for 135, 110, 60, and 40 min at 50, 60, 70, and 80 °C, respectively.

### 3.2. Drying Rate

**Figure 3:**
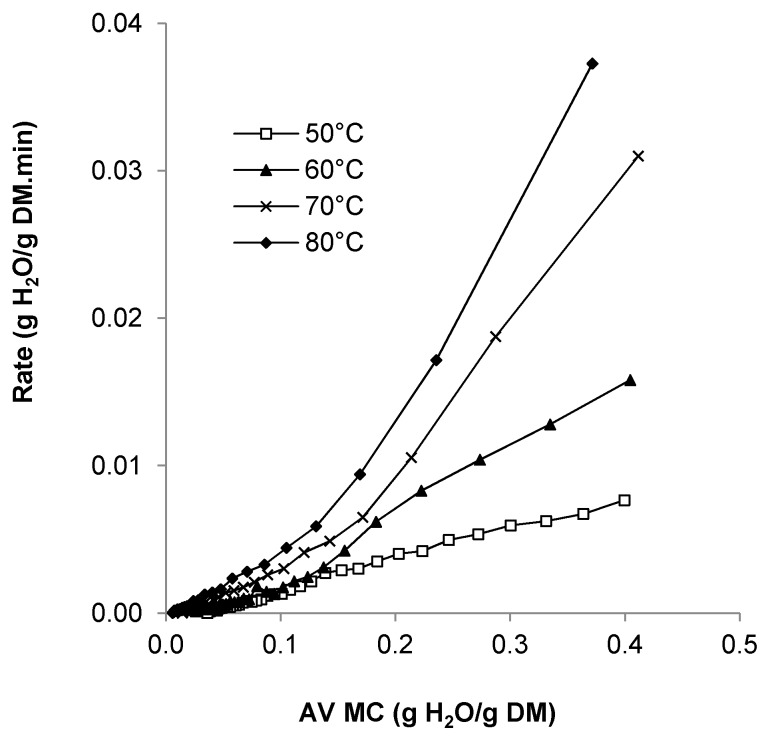
Effect of temperature on drying rate of dried pumpkin seeds, at an air velocity of 2.87 m/s.

The drying rates of pumpkin seeds dried at 50–80 °C are illustrated in Figure 3. Construction of drying rate curves is a common feature of drying studies, as they assist in dryer selection and optimizing of drying conditions [30]. 

Initial drying rates for seeds dried at 50, 60, 70, and 80 °C averaged 0.008, 0.016, 0.031, and 0.037 g H_2_O/g DM/min, respectively. No constant rate period was observed, which means that drying commenced at a moisture level below the critical moisture content and was therefore controlled by diffusion of moisture from within the seeds [30]. This is expected as the seeds were allowed to air dry to an average initial moisture value of 32.71 % (wb) prior to short-term refrigerated storage. For seeds dried at 50 °C, drying rates were found to decline steadily for the duration of the drying run (207.5 min), indicating that drying occurred in one falling-rate period. A first falling drying rate occurs when the surface of the material is no longer saturated with moisture but dry portions of the solid jut out into the air film. Drying involves two processes, namely, the diffusion of moisture within the material to the surface, and the removal of moisture from the surface. The drying rate during this time is proportional to the fraction of surface, which is wet.

For seeds dried at 60 °C and above, drying rates were found to decline rapidly at the higher moisture values, followed by a more gradual decline beyond moisture values of 0.18 g H_2_O/g DM. This represents a change from the first falling-rate period to the second falling-rate period. The second falling rate period begins when the surface is completely dry and the plane of evaporation recedes from the surface, during which internal moisture transfer by diffusion becomes rate limiting [30]. 

Drying rate curves have not been previously reported for pumpkin or squash seeds but drying was reported to occur in the falling-rate period [16,18]. Uddin et al. [16] noted that pumpkin seeds have a high surface area to volume ratio and lose moisture from both surfaces during the fluidized bed drying process compared with loss of moisture primarily from the top surface during tray drying. Drying rates are therefore expected to be higher during fluidized bed drying.

### 3.3. Moisture Ratio

**Figure 4:**
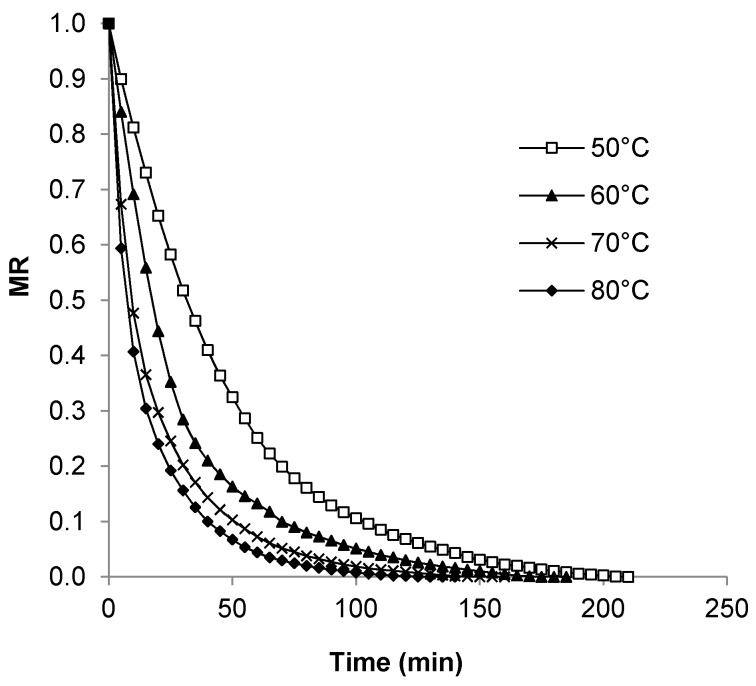
Moisture Ratio (*MR*) curves for pumpkin seeds dried at constant air velocity of 2.87 m/s.

Moisture Ratio (*MR*) curves presented in Figure 4 follow the same trend as the drying curves, highlighting the effect of drying temperature. The higher the temperature, the greater the decline, especially during the first 50 min of drying. The most pronounced temperature effect was seen as the drying temperature increased from 50 to 60 °C. A similar decline in *MR* was seen for salted pumpkin seeds dried at 60–80 °C in a fluidized bed dryer [15]. 

### 3.4. Drying Rate Constants (k) and Effective Diffusion Coefficient (Deff)

**Figure 5:**
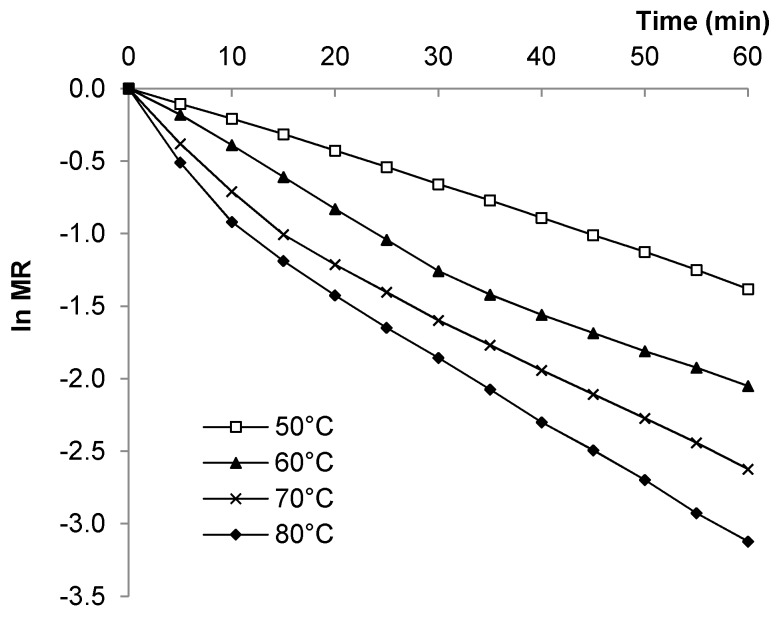
Plots of ln Moisture Ratio versus time for pumpkin seeds dried at constant air velocity of 2.87 m/s.

Plots of ln *MR* versus time (Figure 5) revealed one straight-line segment for seeds dried at 50 °C, but two distinct portions for seeds dried at 60 °C (30 min), 70 °C (15 min), and 80 °C (10 min), representing the transition from first to second falling-rate period. This transition occurred at an average moisture value of 0.18 g H_2_O/g DM. Increasing the drying temperature resulted in a decrease in the time taken to transition from the first falling-rate to the second falling-rate period. 

Drying rate constants (*k*_1_ and *k*_2_) for the two falling rate periods are given in Table 3. As expected, *k*_1_ values increased as drying temperature increased, demonstrating the influence of temperature on moisture loss during the initial stages of drying. Drying rate constants are generally much lower due to the low rate of moisture removal during this stage when the surface of the material is completely dry.

Effective moisture diffusivity values calculated using the drying rate constants are also given in Table 3. Diffusivity values obtained in this study were higher than those reported for the tray drying of salted, hull-less and whole seeds at the same temperatures [13], demonstrating the improved drying efficiency of fluidized bed drying. As expected, the diffusivity values for the second falling-rate period were lower as the amount of water removed during the second falling rate period can be relatively small compared to the first falling rate period. 

The activation energy (*E_a_*) for the first falling rate period was calculated to be 43.9 kJ/mol (*R*^2^ = 0.9817) for the first falling-rate period and 27.9 kJ/mol (*R*^2^ = 0.9763) for the second falling-rate period. A lower activation energy of 33.15 kJ/mol was reported for tray-dried salted, hull-less seeds (40–60 °C) [13], while the activation energy for salted, whole seeds dried at 60–80 °C in a fluidized bed dryer and tray dryer was reported to be 15.0 and 62.12 kJ/mol, respectively [15]. 

### 3.5. Thin Layer Models

**Figure 6:**
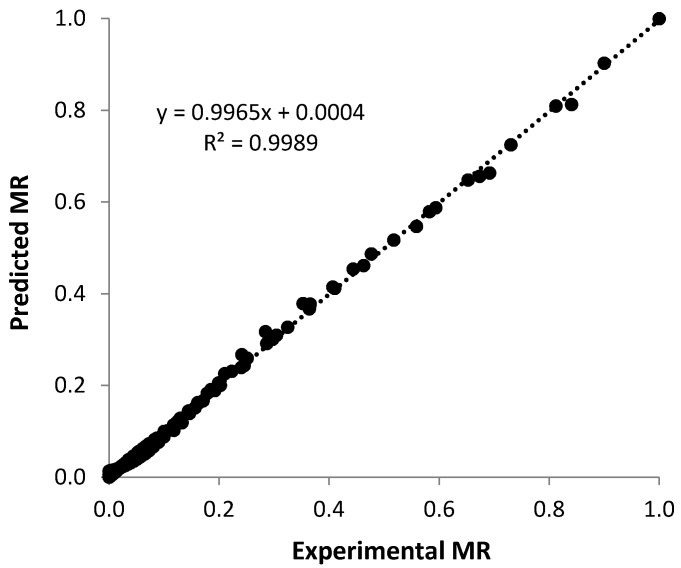
Experimental versus Predicted Moisture Ratio values for pumpkin seeds dried at constant air velocity of 2.87 m/s.

Experimental data for each drying treatment was fitted to twenty-two thin layer models. Table 4 gives the thin layer models that best fit the data for each drying treatment based on the highest *R*^2^ value and lowest value of *RMSE* and *χ*^2^, the approach also used for the tray and fluidized bed drying of pumpkin and squash seeds [13,18]. While model fit varied with drying temperature, the Alibas model could be successfully used (*R*^2^ = 0.9989) as a general model to predict the *MR* data for all temperatures investigated (Figure 6). 

Of the six thin layer models applied to the *MR* data for salted, whole pumpkin seeds dried in a fluidized bed dryer at 60–70 °C (1.8 m/s), the Page model was found to best fit the data [15]. The initial moisture content of the commercially sourced pumpkin seeds used in that study was reported to be 14% (wb), which increased to 25% (wb) after the salting process. The initial moisture content of the pumpkin seeds used in the present study was higher and averaged 32.71% (wb). Of the seven models tested, Chayjan et al. [18] found the two-term model to best fit the data for whole squash seeds dried at 50–80 °C (2.51 m/s and 4.01 m/s) in a fluidized bed dryer. 

### 3.6. Quality Attributes

The results of the assessment of the color attributes and proximate analyses of the pumpkin seed powders are shown in Table 5. There was a slight, visible darkening of the seeds as drying temperature increased from 50 to 80°C and this was reflected in lower L* values (*p* ≤ 0.05) for the seed powders. Redness (+a* values) and yellowing (+b* values) of seeds decreased as temperature was increased from 50 to 80 °C. Uddin et al. [16] reported the discoloration of whole, salted pumpkin seeds at drying temperatures above 70 °C and at higher air velocity settings (3–4 m/s), attributing this to nonenzymatic browning under harsher drying conditions. There was a decrease (*p* ≤ 0.05) in fat content of the seed powders as drying temperature increased from 50 to 70 °C. This could be due to the breakdown of cells resulting in the loss of volatile oils from the seeds at the higher temperatures. Seed powders were high in both crude fiber and protein, with values averaging 0.23 and 0.29 g/g DM, respectively. Values for crude fiber and protein content of pumpkin seeds reported in the literature vary widely, with the results of this study falling within the range of values reported [4,10,12,31].

There is a wide variation in the literature on recommendations for drying pumpkin seeds, depending on the type and treatment of seeds (with or without hull, raw or salted, dried or roasted) as well as the final application (as a snack or for use as a seed powder). A commonly used oven drying temperature for the production of seed powder is 60 °C, for various periods (6–24 h) [2,4,10]. Based on the present study, pumpkin seeds can be dried for 110 min (1.83 h) to a final moisture content of 4–6% (wb), highlighting the rapid drying that can be achieved using the fluidized bed drying method. Based on fast drying rates, Jittanit [15] selected salted, whole pumpkin seeds dried in a fluidized bed dryer at 80 °C (1.8 m/s), reporting that the scores were slightly lower for appearance, color, aroma, and taste, but higher with respect to texture and overall liking compared with commercial samples. They did, however, note that seeds dried in a tray drier at the same temperature were generally preferred over samples dried in a fluidized bed drying.

Roasting of pumpkin seeds prior to consumption is a common practice, which helps in the formation of flavor, color, texture, and therefore, overall palatability. Some researchers have recommended roasting (100–120 °C, 0.25–24 h) of whole, dehulled or defatted seeds in the production of seed flour prior to use in cookies and breads [3,5]. The changes that occur during the roasting process are mainly related to nonenzymatic browning [32]. In the present study, seeds dried at 70 and 80 °C developed roasted odors. Kuar [11] roasted whole, sun-dried seeds at 70 °C (15–20 min) prior to grinding seeds into a powder to be used as a protein supplement in cookies.

It was determined that 92.7, 96.0, 94.3, and 98.5% of the pumpkin powder dried at 50–80 °C had a particle size greater than 3.36 mm, and it was observed that the seeds dried at the higher temperatures (70 and 80 °C) were more brittle and therefore easier to grind. Uddin et al. [16] recommended that whole, salted pumpkin seeds be dried at 70 °C at an air velocity of 2 m/s, reporting that harsher drying conditions resulted in undesirable changes in color and texture of the seeds. The physical properties of the seed powders were not significantly (*p* ≤ 0.05) affected by drying temperature, with bulk and tapped density values averaging 283.2 ± 8.14 and 393.55 ± 8.93 kg/m3, respectively. Carr Index and Hausner Ratio of the ground seeds averaged 0.28 ± 0.02 and 1.39 ± 0.04, respectively. The Carr Index is an indication of the flowability of a powder, with a value of 28.4% being within the range for ‘fair’ flowability (20%–35%). The Hausner ratio is an indication of the cohesiveness of the powder and a value of 1.40 represents an ‘intermediate’ cohesiveness (1.2–1.4). Bulk density and water absorption values for flour made from de-hulled fluted pumpkin seeds averaged 0.20 g/mL 3.4 g H_2_O/g powder, respectively [32]. Lower water absorption values (2.58 and 1.89 g H_2_O/g powder) were reported previously for whole dried seed and powder [4,12]. The physical properties investigated are a direct function of the particle size and vary across previous works, depending on the end use of the seed powder [2,3,4,5,6,8,11].

## 4. Conclusions

Drying of pumpkin seeds for the purpose of making pumpkin seed powders was successfully carried out in a fluidized bed dryer at 50–80 °C. A moisture content of 4–6% (wb) could be achieved by drying seeds for 135, 110, 60 and 40 min at 50, 60, 70, and 80 °C, respectively. The drying behavior was described by the corresponding drying curves and the Alibas model was found to successfully predict the Moisture Ratio (*MR*) data for seeds dried at all temperatures. Increasing the temperature increased the drying rate and drying occurred in the falling-rate period only, with drying at 60–80 °C occurring in two distinct periods.

Dried seed powders were found to have high protein, fiber and fat contents. Increasing the temperature from 50 to 80 °C had an impact on the lightness of the seed powders, but no browning was observed. Seeds developed a roasted odor at temperatures above 60 °C. This flavor development is considered desirable in some applications. The seed powder could be easily ground into a powder and can therefore be suitable for incorporation into baked goods, where the particle size can be adjusted for the particular application. Further work can investigate the defatting of the dried seeds, depending on the end-use of the powder.

## Figures and Tables

**Table 1:** Thin layer drying models.

Model Name	Equation
Newton	*MR = exp (−Kt)*
Page	*MR = exp(−Kt^n^)*
Modified Page	*MR = exp(−Kt)^n^*
Henderson and Pabis	*MR = a exp (−Kt)*
Modified Henderson and Pabis	*MR = a exp (−Kt) + b exp (−gt) + c exp (−ht)*
Logarithmic	*MR = a exp (−Kt) + c*
Two-Term	*MR = a exp (−K*_0_* t) + b exp (−K*_1_* t)*
Two-Term Exponential	*MR = a exp (−K t) + (*1*−a) exp (−K a t)*
Wang & Singh	*MR = *1* + at + bt* ^2^
Verma	*MR = a exp(−kt)+(*1*−a) exp(−gt)*
Hii	*MR = a exp(−Kt^n^) + c exp(−gt^n^)*
Midilli	*MR = a exp (−Kt^n^) + b t*
Weibull distribution	*MR = a−b exp (−Kt^n^)*
Diffusion approach	*MR = a exp(−Kt) + (*1*−a) exp(−Kbt)*
Aghbashlo et al.	*MR = −K*_1_*t/(*1* + K*_2_*t)*
Logistic	*MR = a_0_/((*1* + a exp (Kt))*
Jena and Das	*MR = a exp (−Kt + b t^*1/2*^) + c*
Demir et al.	*MR = a exp (−Kt^n^) + c*
Alibas	*MR = a exp (−Kt^n^ + b t) + g*

**Table 2:** Time taken for dried pumpkin seeds to attain equilibrium, equilibrium moisture content and water activity values of dried seeds, at a fixed velocity of 2.87 m/s.

Parameter	Drying Temperature			
	50 °C	60 °C	70 °C	80 °C
Time to Equilibrium (min)	207.5 ± 2.5 ^a^	170.0 ± 5.0 ^b^	147.5 ± 2.5 ^c^	122.5 ± 7.5 ^d^
Moisture Content (g H_2_O/g DM)	0.035 ± 0.0004 ^a^	0.026 ± 0.0005 ^b^	0.015 ± 0.0005 ^c^	0.006 ± 0.001 ^d^
Water activity (a_w_)	0.418 ± 0.008 ^a^	0.398 ± 0.008 ^a^	0.267 ± 0.011 ^b^	0.267 ± 0.019 ^b^

Values are means ± SEM, *n* = 2 per treatment group. ^a–d^ Means in a row without a common superscript letter differ (*p* < 0.05) as analyzed by one-way ANOVA and the LSD test.

**Table 3:** Drying rate constants (*k*) and effective moisture diffusivity values (*D_eff_*) for pumpkin seeds dried in a fluidized bed dryer at a constant velocity of 2.87 m/s.

Temperature (°C)	Drying Rate Constant, *k* (1/min)				Effective Moisture Diffusivity, *D_eff_* (m^2^/s)	
	*k*_1_	*R* ^2^	*k*_2_	*R* ^2^	*D*_1_	*D*_2_
**50**	0.0226 ^a^	0.9994	-	-	4.68 × 10^−10^	-
**60**	0.0423 ^b^	0.9985	0.0241 ^a^	0.9963	8.76 × 10^−10^	1.25 × 10^−10^
**70**	0.0673 ^c^	0.9969	0.0349 ^b^	0.9993	13.94 × 10^−10^	1.81 × 10^−10^
**80**	0.0900 ^d^	0.9961	0.0426 ^c^	0.9996	18.63 × 10^−10^	2.21 × 10^−10^

*n* = 2 per treatment group. ^a–d^ Means in a column without a common superscript letter differ (*p* < 0.05) as analyzed by one-way ANOVA and the LSD test. L = half thickness = 0.00175 m.

**Table 4:** Thin layer model fit and constants for pumpkin seeds dried at a) 50 °C, b) 60 °C c) 70 °C and d) 80 °C at a constant air velocity of 2.87 m/s.

a) 50 °C, 2.87 m/s													
Model	Model Constants obtained by curve fitting										R^2^	RMSE	χ^2^
	k	n	a	b	c	g	k_1_	k_2_	a_0_	h			
Alibas	1.2780	1.0005	1.0047	1.2581	-	−0.0022	-	-	-	-	0.9999	0.004237	0.000020
Logistic	0.0240	-	8.2151	-	-	-	-	-	9.2575	-	0.9999	0.004534	0.000022
Aghbashlo et al.	-	-	-	-	-	-	0.0217	−0.0005	-	-	0.9998	0.005065	0.000027
**b) 60 °C, 2.87 m/s**													
**Model**	**Model Constants**										**R^2^**	**RMSE**	**χ^2^**
	**k**	**n**	**a**	**b**	**c**	**g**	**k_1_**	**k_2_**	**a_0_**	**h**			
Aghbashlo et al.	-	-	-	-	-	-	0.0429	0.0037	-	-	0.9986	0.012437	0.000163
Alibas	2.8099	0.9987	1.0116	2.7577	-	0.0102	-	-	-	-	0.9982	0.014473	0.000241
**c) 70 °C, 2.87 m/s**													
**Model**	**Model Constants**										**R^2^**	**RMSE**	**χ^2^**
	**k**	**n**	**a**	**b**	**c**	**g**	**k_1_**	**k_2_**	**a_0_**	**h**			
Alibas	0.5837	0.9668	1.0439	0.4725	-	−0.0405	-	-	-	-	0.9998	0.004460	0.000023
Aghbashlo et al.	-	-	-	-	-		0.0756	0.0112	-	-	0.9980	0.013693	0.000206
**d) 80 °C, 2.87 m/s**													
**Model**	**Model Constants**										**R^2^**	**RMSE**	**χ^2^**
	**k**	**n**	**a**	**b**	**c**	**g**	**k_1_**	**k_2_**	**a_0_**	**h**			
Modified Henderson & Pabis	0.2153	-	0.4646	0.1883	0.3472	0.0416	-	-	-	0.0416	1.0000	0.001141	0.000002
Alibas	0.3455	0.9090	1.0688	0.2007	-	−0.0681	-	-	-		0.9999	0.002719	0.000009
*Aghbashlo* et al.	*-*	*-*	*-*	*-*	*-*	*-*	*0.0987*	*0.0154*	*-*	*-*	*0.9978*	*0.014857*	*0.000238*

*R*^2^, Coefficient of Determination; *RMSE*, Root Mean Square Error; *χ*^2^, Chi-Square statistic.

**Table 5:** Comparison of selected quality attributes and proximate composition of pumpkin seed powders obtained from seeds dried at 50 to 80 °C.

Quality Attribute	Temperature (°C)			
	50 °C	60 °C	70 °C	80 °C
*L* ^*^	64.21 ± 0.12 ^a^	63.13 ± 0.32 ^b^	57.43 ± 0.51 ^c^	54.94 ± 0.15 ^d^
*a* ^*^	3.04 ± 0.01 ^a^	2.90 ± 0.05 ^a^	2.58 ± 0.50 ^a^	2.83 ± 0.02 ^b^
*b* ^*^	18.98 ± 0.04 ^a^	18.40 ± 0.53 ^a^	18.07 ± 0.13 ^a^	17.93 ± 0.16 ^b^
*Hue* (°)	80.89 ± 0.04	81.04 ± 0.40	81.85 ± 1.76	81.02 ± 0.12
Fat (g/g DM)	0.35 ± 0.004 ^a^	0.34 ± 0.010 ^a^	0.32 ± 0.002 ^b^	0.32 ± 0.004 ^b^
Crude Fiber (g/g DM)	0.22 ± 0.006	0.24 ± 0.010	0.23 ± 0.020	0.22 ± 0.004
Protein g/g DM	0.27 ± 0.004 ^a^	0.29 ± 0.010 ^ab^	0.29 ± 0.013 ^ab^	0.31 ± 0.003 ^b^

*n* = 3 per treatment group. ^a–d^ Means in a column without a common superscript letter differ (*p* < 0.05) as analyzed by one-way ANOVA and the LSD test.

## Nomenclature

*E_a_*	Activation energy (KJ/mol)
*D_eff_*	Effective Diffusion coefficient (m^2^/s)
*DM*	Dry matter
*k*_1_ *, k*_2_	Drying rate constants (1/min) obtained from slopes of plot of ln *MR* versus time
*L*	Half-thickness of leaf (m)
*M*	Moisture content (g H_2_O/g DM) at time = *t*
*M*_0_	Initial Moisture Content (g H_2_O/g DM)
*M_e_*	Equilibrium Moisture Content (g H_2_O/g DM)
*MR*	Moisture Ratio
*R*	Gas constant (8.314 J/mol.K)
*R* ^2^	Coefficient of Determination
*RMSE*	Root Mean Square Error
*t*	Time (min)
*T*	Temperature (°C)
*wb*	Wet basis
*K*, *a*, *b*, *c*, *g*, *K*_1_, *K*_2_, *a*_0_, *h*	Thin layer model constants obtained by curve fitting
*χ* ^2^	Chi-Square Statistic

## References

[1] United States Department of Agriculture (USDA)—Agricultural Research Service; National Nutrient Database for Standard Reference Legacy Release 12014, Seeds, pumpkin and squash seed kernels, dried. https://ndb.nal.usda.gov/ndb/foods.

[2] Giami S.Y., Isichei I. (1999). Preparation and properties of flours and protein concentrates from raw, fermented and germinated fluted pumpkin (*Telfairia occidentalis* Hook) seeds. Plant Food Hum. Nutr..

[3] Hamed S.Y., Hassan N.M.E., Hassan A.B., Eltayeb M.M., Babiker E.E. (2008). Nutritional evaluation and physiochemical properties of processed pumpkin (*Telfairia occidentalis* Hook) seed flour. Pak. J. Nutr..

[4] Nyam K.L., Lau M., Tan C.P. (2013). Fibre from pumpkin (*Cucurbita pepo* L.) seeds and rinds: physio-chemical properties, antioxidant capacity and application as bakery product ingredients. Mal. J. Mutr..

[5] Saraswathi D., Renu R., Maloo S. (2018). Development and quality evaluation of pumpkin seeds and flaxseeds powder incorporated biscuits. Int. J. Food Sci. Nutr..

[6] Dhiman A.K., Bavita K., Attri S., Ramachandran P. (2018). Preparation of pumpkin powder and pumpkin seed kernel powder for supplementation in weaning mix and cookies. Intl. J. Chem. Studies.

[7] Bello F.A., Udo I.I., Mbak D.L. (2017). Proximate composition and functional properties of sprouted sorghum (*Sorghum bicolor*) and defatted fluted pumpkin seed (*Telfairia occidentalis*) flour blends. Am. J. Innov. Res. Appl. Sci..

[8] Atuonwu A., Akobundu E. (2010). Nutritional and sensory quality of cookies supplemented with defatted pumpkin (*Cucurbita pepo*) seed flour. Pak. J. Nutr..

[9] Ardabili A.G., Farhoosh R., Khodaparast M.H.H. (2011). Chemical composition and physicochemical properties of pumpkin seeds (*Cucurbita pepo* Subsp. pepo Var. Styriaka) grown in Iran. J. Agr. Sci. Tech..

[10] Fedha M.S. (2008). Physicochemical Characterization and Food Application Potential of Pumpkin (*Cucurbita* sp.) Fruit and Seed Kernel Flours. Master’s Thesis.

[11] Kaur M., Sharma S. (2017). formulation and nutritional evaluation of cookies supplemented with pumpkin seed (*Curcubita Moschata*) flour. Chem. Sci. Rev. Lett..

[12] Sharma G.S., Lakhawat S. (2017). Development, Quality Evaluation and Acceptability of Pumpkin Seed Flour Incorporated in Gravy. J. Nutr. Food Sci..

[13] Sacilik K. (2007). Effect of drying methods on thin-layer drying characteristics of hull-less seed pumpkin (*Cucurbita pepo* L.). J. Fd. Eng..

[14] Kaur M. (2017). Development and Nutritional Evaluation of Pumpkin Seed (*Cucurbita moschata*) Supplemented Products. Master’s Thesis.

[15] Jittanit W. (2011). Kinetics and temperature dependent moisture diffusivities of pumpkin seeds during drying. Kasetsart J. Nat. Sci..

[16] Uddin Z., Suppakul P., Boonsupthip W. (2016). Effect of air temperature and velocity on moisture diffusivity in relation to physical and sensory quality of dried pumpkin seeds. Dry Technol..

[17] Bansal P.K., Chung K.Y., Hui Y.H., Clary C., Farid M.M., Fasina O.O., Noomhorm A., Welti-Chanes J. (2008). Food Drying Equipment, Chapter 15. Food Drying Science and Technology Microbiology, Chemistry, Applications.

[18] Chayjan R.A., Salari K., Abedi Q., Ali Akbar S. (2013). Modeling moisture diffusivity, activation energy and specific energy consumption of squash seeds in a semi fluidized and fluidized bed drying. J. Food Sci. Technol..

[19] Ramsumair S. (2018). Fluidized Bed Drying of Pumpkin (Cucurbita sp.) Seeds. Master’s Thesis.

[20] Mujaffar S., Lee Loy A. (2016). Drying kinetics of microwave-dried vegetable amaranth (*Amaranthus dubius*) leaves. Food Res. Int..

[21] (1999). Official Methods of Analysis of AOAC INTERNATIONAL (1999).

[22] (2000). Official Methods of Analysis of AOAC INTERNATIONAL (2000).

[23] Roongruangsri W., Bronlund J.E. (2016). Effect of air-drying temperature on physico-chemical, powder properties and sorption characteristics of pumpkin powders. Food Res. Int..

[24] Dirim S.N., Çalıskan G.K. (2012). Determination of the effect of freeze drying process on the production of pumpkin (Cucurbita moschata) puree powder and the powder properties. J. Food.

[25] Mujaffar S., Sankat C.K. (2005). The air drying behavior of shark fillets. Can. Biosyst. Eng..

[26] Mujaffar S., Sankat C.K. (2015). Modeling the drying behavior of unsalted and salted catfish (*Arius* sp.) slabs. J. Food Process. Preserv..

[27] Ertekin C., Firat M.Z. (2017). A comprehensive review of thin-layer drying models used in agricultural products. Crit. Rev. Food Sci. Nutr..

[28] Hyams D.G. Hyams Development—CurveExpert Development. http://www.curveexpert.net..

[29] Assaad H.I., Hou Y., Zhou L., Carroll R.J., Wu G. (2014). Rapid publication-ready MS-Word tables for two-way ANOVA. SpringerPlus.

[30] Sablani S.S., Rahman M.S., Hui Y.H., Clary C., Farid M.M., Fasina O.O., Noomhorm A., Welti-Chanes J. (2008). Fundamentals of Food Dehydration, Chapter 1. Food Drying Science and Technology Microbiology, Chemistry, Applications.

[31] Giami S.Y., Bekebain D.A. (1992). proximate composition and functional properties of raw and processed full-fat fluted pumpkin (*Telfairia occidentalis*) seed flour. J. Sci. Food Agric..

[32] Buckholz L., Daun H., Stier E. (1980). Influence of roasting time on sensory attributes of fresh roasted peanuts. J. Food Sci..

